# MULTIDOMAIN INTERVENTION TO IMPROVE COGNITION IN OLDER ADULTS: NEW RESULTS AND FUTURE CHALLENGES

**DOI:** 10.1093/geroni/igae098.2185

**Published:** 2024-12-31

**Authors:** Louis Bherer

**Affiliations:** Chair

## Abstract

Decades of research have supported the notion that physical exercise and cognitive training can both help enhance cognitive performances in older adults. More recent studies have investigated whether these two domains of intervention could have synergistic effects, however results are equivocal. Moreover, there is an important heterogeneity among studies with regards to intervention modality within domain (e.g., memory or attention training as cognitive intervention; aerobic, resistance or combined physical exercise) and studied population (MCI vs. healthy aging). This symposium will discuss recent findings from innovative interventions ranging from within-domain attention training to multi-domain interventions that combine cognitive training or cognitive stimulation with or without physical exercise components. Issue of interindividual differences and the need to better individualized intervention as well as diversity in targeted population will also be discussed. Presentations will also address future challenges in combined intervention studies.

